# 352. COVID-19 Not a Risk Factor of Alopecia Areata: Results of a National Cohort Study in South Korea

**DOI:** 10.1093/ofid/ofab466.553

**Published:** 2021-12-04

**Authors:** Jeehyun Kim, Kwan Hong, Sujin Yum, Raquel Elizabeth Gomez Gomez, Byung Chul Chun

**Affiliations:** 1 Korea University, Seoul, Seoul-t’ukpyolsi, Republic of Korea; 2 Korea University College of Medicine, Seoul, Seoul-t’ukpyolsi, Republic of Korea

## Abstract

**Background:**

There have been approximately 158 million coronavirus disease 2019 (COVID-19) pandemic survivors worldwide by June 9, 2021. As a result, concerns about hair loss in COVID-19 patients have emerged among dermatologists. However, most of extant literature have limited implications by relying on cross-sectional studies with restricted study subjects without control group. Therefore, our study aims to investigate the risk of developing alopecia areata (AA) among COVID-19 patients in South Korea using adequate control based on national representative data.

**Methods:**

We used the National Health Insurance Service (NHIS) COVID‐19 cohort database, comprising COVID‐19 patient and control group, all of whom were diagnosed from January 1, 2020 to June 4, 2020. Patients were defined as individuals who were confirmed as COVID‐19 positive, regardless of disease severity. Controls were defined as whom confirmed as COVID‐19 negative. People with a history of AA during the period 2015–2019 were excluded. The primary endpoint was a new diagnosis of AA (ICD-10-CM-Code: L63). Adjusted incidence rate ratio (IRR) of developing AA was estimated using log-link Poisson regression model based on incidence density of case and control group. The model adjusted for (1) age and sex (2) demographic variables (age, sex, place of residence, and income level). Statistical significance was set at p< 0.05.

**Results:**

A total of 226,737 individuals (7,958 [3.5%] cases and 218,779 [96.5%] controls) were included in the final analysis. There were more females than males, both in test positives and negatives at 59.9% and 52.3%, respectively. The largest test positive population was those in age group 20 to 29 years (25.5%),. The test negatives had the largest population in age group 30 to 39 years (17.1%). The ratio of newly diagnosed AA was 18/7,958 (0.2%) in cases and 195/218,779 (0.1%) in controls. IRRs of COVID-19 patients having newly diagnosed AA compared to controls were 0.78 (0.48‒1.27) when age and sex were adjusted for, and 0.60 (0.35‒1.03) when all demographic variables were adjusted for.

Flowchart of study subject selection

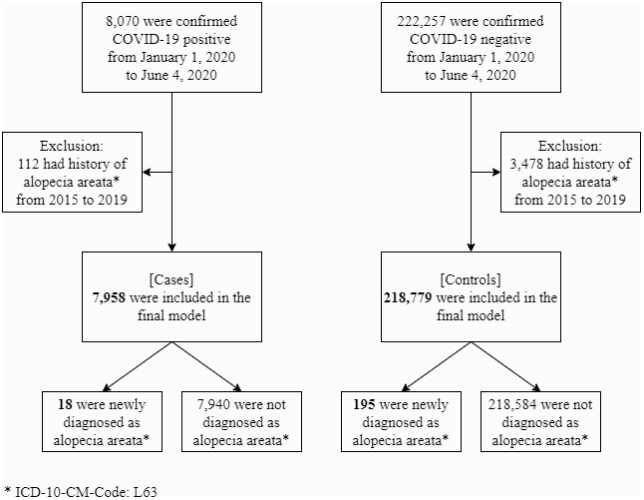

**Conclusion:**

Diagnosis of COVID-19 was not significantly associated with development of AA even after appropriately adjusting for covariates.

**Disclosures:**

**All Authors**: No reported disclosures

